# Survival prediction models for people living with HIV based on four machine learning models

**DOI:** 10.1038/s41598-025-16479-3

**Published:** 2025-08-25

**Authors:** Qiong Cai, Lanting Yang, Yulong Ling, Wei Pan, Qing Zhong, Chunjie Wang, Xilong Pan

**Affiliations:** 1 Department of Social Medicine and Health Education, School of Public Health, Peking University, 38 College Road, Haidian District, Beijing, 100191 China; 2 Faculty of Arts, The University of Melbourne, VIC, Melbourne, VIC 3052 Australia; 3 School of Computer Science and Technology, Harbin Institute of Technology, Weihai, 264209 China

**Keywords:** HIV, AIDS, Machine learning, Artificial intelligence, Prediction models, Diseases, Medical research, Risk factors

## Abstract

Although antiretroviral therapy has prolonged the lifespan of people living with HIV, significant variations still exist in survival rates and risk factors among these people. This study compares the performance of the Cox proportional hazard models with four machine learning models in predicting the survival of people living with HIV, analyzing the survival factors among them, thereby assisting medical decision-making. We collected data on 676 people living with HIV from the Chinese Center for Disease Control and Prevention. Significant variables (*p* < 0.05) were identified using Cox univariate analysis. Using a random number method, the data were split into a training set (473 cases) and a test set (203 cases) in a 7:3 ratio. We employed the Cox proportional hazard model and four classification machine learning models, including eXtreme Gradient Boosting, Random Forest, Support Vector Machine, and Multilayer Perceptron, to develop survival prediction models for people living with HIV. The predictive performance of these models was evaluated based on accuracy, precision, recall, F1-score, area under the receiver operating characteristic curve (AUC), and calibration curves, and the best model was selected based on these metrics. The average age of diagnosis among the sample participants was 56.63 years (SD = 17.53). Considering the performance of both the training and testing cohorts, the Random Forest classifier emerged as the model with the best predictive performance, with an AUC of 0.912, an Accuracy of 0.862, a Precision of 0.794, a Recall of 0.562, and an F1 score of 0.659. Random Forest was followed by the Support Vector Machine, the eXtreme Gradient Boosting, Multilayer Perceptron, and the Cox proportional hazard model performed similarly. The predictive performance of machine learning models surpasses traditional Cox proportional hazard models. In China, the Random Forest model can be considered for analyzing and predicting the survival rates of people living with HIV.

**Supplementary Information:**

The online version contains supplementary material available at 10.1038/s41598-025-16479-3.

## Introduction

Acquired Immunodeficiency Syndrome(AIDS) is a highly dangerous systemic infectious disease caused by the Human Immunodeficiency Virus (HIV)^1^. According to statistics from the World Health Organization, millions of people are infected with HIV annually, and HIV infection remains one of the most severe public health challenges globally^2,3^. Although the development of Antiretroviral Therapy (ART) has extended the lifespans of people living with HIV (PLWH)^4^variations in survival rates still exist under the influence of various factors^5^.

ART could slow the progression of HIV disease, but it does not eradicate HIV^6^. Henan Province in China was one of the first regions to initiate ART treatment, resulting in an aging population among PLWH due to early treatment^7^. Although PLWH significantly benefit from ART^8^compared to their age-matched peers without HIV infection, they face a higher risk of multimorbidity levels of diseases^9,10^. Therefore, their survival outcomes warrant further discussion and analysis. Previous studies primarily used analytical methods such as the Kaplan-Meier curve^11^log-rank test^12^multivariate logistic regression^13^and Cox proportional hazard regression analysis^14^. Some studies have proposed more flexible and advanced analytical models like the Cox-Aalen model^15^ and the semiparametric linear transformation model^16^but most are still based on innovations and modifications of the Cox proportional hazard model. Currently, the mainstream analytical pathway continues to be univariate analysis combined with Cox proportional hazard regression analysis^17^. Although traditional Cox proportional hazard models have good interpretability, they may be limited when dealing with complex, high-dimensional, or non-linear data.

Machine learning is the use of algorithms to parse processed data, make predictions about events, and continuously learn and self-improve models^18^. Although there are deep survival models available to improve prediction accuracy, these models require significant data and computational resources and have black-box properties that are difficult to interpret. Machine learning models are a relatively balanced approach, with a level of complexity between traditional Cox proportional hazards regression models and deep survival models. While providing good predictive performance, machine learning models can also provide some interpretability. Machine learning possesses powerful data processing and pattern recognition capabilities, demonstrating high accuracy in survival prediction^19^. To date, machine learning techniques have been widely applied in survival analysis in various disease domains such as cancer^20^chronic kidney disease^21^and connective tissue disease^22^. However, research on the prognosis and survival analysis of PLWH remains limited, primarily focusing on disease forecasting and health management^23^. Henan Province is a typical HIV-infected city, and the distribution of PLWH in Hebi is similar to that of Henan Province, which, to a certain extent, can reflect the survival of PLWH in China. This study aims to build a survival prediction model for PLWH after diagnosis, based on machine learning models using data from PLWH in Hebi, explore the factors affecting the survival of PLWH, and assess the value of machine learning models in predicting the survival of PLWH.

## Materials and methods

### Study subjects

This study is a retrospective analysis. From the antiretroviral treatment information management system of the Chinese Center for Disease Control and Prevention, historical reports and follow-up records of PLWH in Hebi, Henan Province, were selected from January 1, 2003, to December 31, 2023, with the data accessed on Aug 20, 2024. Inclusion criteria: (1) People living with HIV. The diagnosis of HIV/AIDS is based on laboratory tests, combined with clinical manifestations and referenced epidemiological data; (2) Age ≥ 18 years. Exclusion criteria: (1) Missing or erroneous data on variables (missing or misplaced remaining variables due to errors in the entry of occupational variables, 16 cases); (2) Presence of other severe diseases or complications; (3) Non-residents or non-long-term residents(< 6 months) of Hebi, Henan Province. A total of 692 individuals were sampled, and finally, 676 individuals who met the criteria were enrolled in the study. This research was approved by the Ethics Committee of the Hebi Center for Disease Control and Prevention(license number: 2024-003). Because the study did not involve anyone’s private information, the ethics committee waived the requirement for informed consent. The study was consistent with the Declaration of Helsinki principles.

### Study variables

In this study, the observation start time is the date of diagnosis of HIV infection for the subjects, and the observation end time is December 31, 2023. Failure events of the study are all-cause mortality in HIV, with censored events including withdrawal or survival at the end of follow-up. The data content includes the following for PLWH: (1) Basic information: age at diagnosis, gender, marital status, educational level, history of venereal disease, routes of infection; (2) Clinical indicators: CD4^+^T cell count, CD8^+^T cell count, HIV viral load; (3) Treatment information: Whether or not receiving ART?, duration of treatment; (4) Follow-up records: survival status, survival time, cause of death, etc. Because the missing values for three variables, CD8^+^ T-cell count, HIV viral load, and duration of treatment, exceeded 20% of the sample size, data interpolation tended to affect the accuracy of the model. Therefore, we removed these variables. In addition, 16 cases of sample data were missing or structurally misplaced for the remaining variables due to errors in the entry of occupational variables. The sample was excluded from the study because the misaligned data could not be repaired, and the missing mechanism was completely random. There were no missing values for the other variables, and all variables were categorical (Table S1).

### Feature selection and survival analysis

Using a random number method, all data were divided in a 7:3 ratio into a training cohort of 473 cases and a testing cohort of 203 cases. The training cohort was used for model building, and the testing cohort was used for model validation. The Cox proportional hazard model is typically used to describe the impact of multiple characteristics, which do not change over time, on the mortality rate at a given moment. The study utilized the Kaplan-Meier method to determine whether the study variables met the assumptions of the proportional hazard model and to test whether differences exist between the two groups of PLWH. Upon satisfying the assumptions of the proportional hazard model, the study variables were subjected to between-group analysis of variance and Cox univariate analysis of variance, which resulted in the screening of clinically significant variables. Statistically significant variables (*p* < 0.05) were included in the multivariate Cox proportional hazard model with two-sided P-values, α = 0.05. A nomogram was used to visually interpret the model.

### Construction and interpretation of machine learning models

Variables that showed statistically significant differences in the between-group analysis of variance and Cox univariate analysis were selected as inputs for the models. Based on the training cohort, four types of machine learning models were established: eXtreme Gradient Boosting (XGBoost), Random Forest (RF), Support Vector Machine (SVM), and Multilayer Perceptron (MLP). Grid search combined with 5-fold cross-validation is used to determine the optimal hyper-parameters for each model, and the models are compared based on the 5-fold cross-validation to select the best model. Furthermore, Shapley Additive exPlanations (SHAP) values were calculated based on the optimal model to provide a visual interpretation of the machine learning model. The hyper-parameter tuning is shown in Table S2.

### Statistical methods

Data analysis was conducted using R, version 4.4.1. Quantitative data were expressed in numbers and percentages, and comparisons between groups were made using the Chi-square test or the exact probability method. Cox univariate regression analysis was used to compare differences between two groups and to select variables, with *p* < 0.05 considered statistically significant. The multivariate Cox proportional hazard regression model was constructed using R, version 4.4.1, and the correlation analysis, variance inflation factors, and machine learning models were built using Python 3.12. Additionally, we built the depsurv model, the dephit model, and the random survival forest model using Python 3.12 to complement the study.

## Results

### General analysis

Based on the inclusion and exclusion criteria, a total of 676 cases were enrolled in this study. 529 cases were male and 147 cases were female, with a male-to-female ratio of 3.6:1. The youngest PLWH was 18 years old, and the oldest was 99 years old. The majority of diagnoses occurred in PLWH aged 40 and above, accounting for 81.1% of cases. The average age at diagnosis is 56.63 ± 17.53 years. Refer to Table 1.

**Table 1:** Demographic and clinicopathological characteristics of PLWH.

Variable	Surviving (*n* = 507)		Dead (*n* = 169)		$\:{\upchi\:}2$	*p* value
	No. of people	%	No. of people	%		
Onset_age_class						
< 40	123	24.26	5	2.96	151.797	< 0.001
≥ 40, < 60	254	50.10	30	17.75		
≥ 60	130	25.64	134	79.29		
Gender						
Male	384	75.74	145	85.80	14.360	0.006
Female	123	24.26	24	14.20		
Marital_Status						
Unmarried	97	19.13	25	14.79	45.922	< 0.001
Married with Spouse	291	57.40	57	33.73		
Discovered or Widowed	119	23.47	87	51.48		
Education_Level						
Illiterate	31	6.11	39	23.08	65.457	< 0.001
Junior High School and Below	321	63.31	117	69.23		
High School/Technical Secondary School	89	17.55	8	4.73		
College and above	66	13.02	5	2.96		
Occupation						
Unemployed	118	23.27	21	12.43	23.314	< 0.001
Farmer	292	57.59	132	78.11		
Laborer	70	13.81	8	4.73		
Business service and others	27	5.33	8	4.73		
Infection_pathway						
Homosexual transmission	202	39.84	11	6.51	48.779	< 0.001
Heterosexual transmission	268	52.86	140	82.84		
Bloodborne transmission	31	6.11	15	8.88		
Other routes	6	1.18	3	1.78		
Venereal_history						
None	381	75.15	145	85.80	8.510	0.005
Yes	25	4.93	9	5.33		
Unknown	100	19.72	15	8.88		
Last_CD4_result						
< 200	48	9.47	62	36.69	146.313	< 0.001
≥ 200, < 400	128	25.25	64	37.87		
≥ 400	331	65.29	43	25.44		
ART_treatment_status						
No	24	4.73	53	31.36	198.899	< 0.001
Yes	483	95.27	116	68.64		

^a^‘ART_treatment_status’ refers to whether the last follow-up visit forward received ART for no less than six months.

### Cox proportional hazard model

**Fig. 1:**
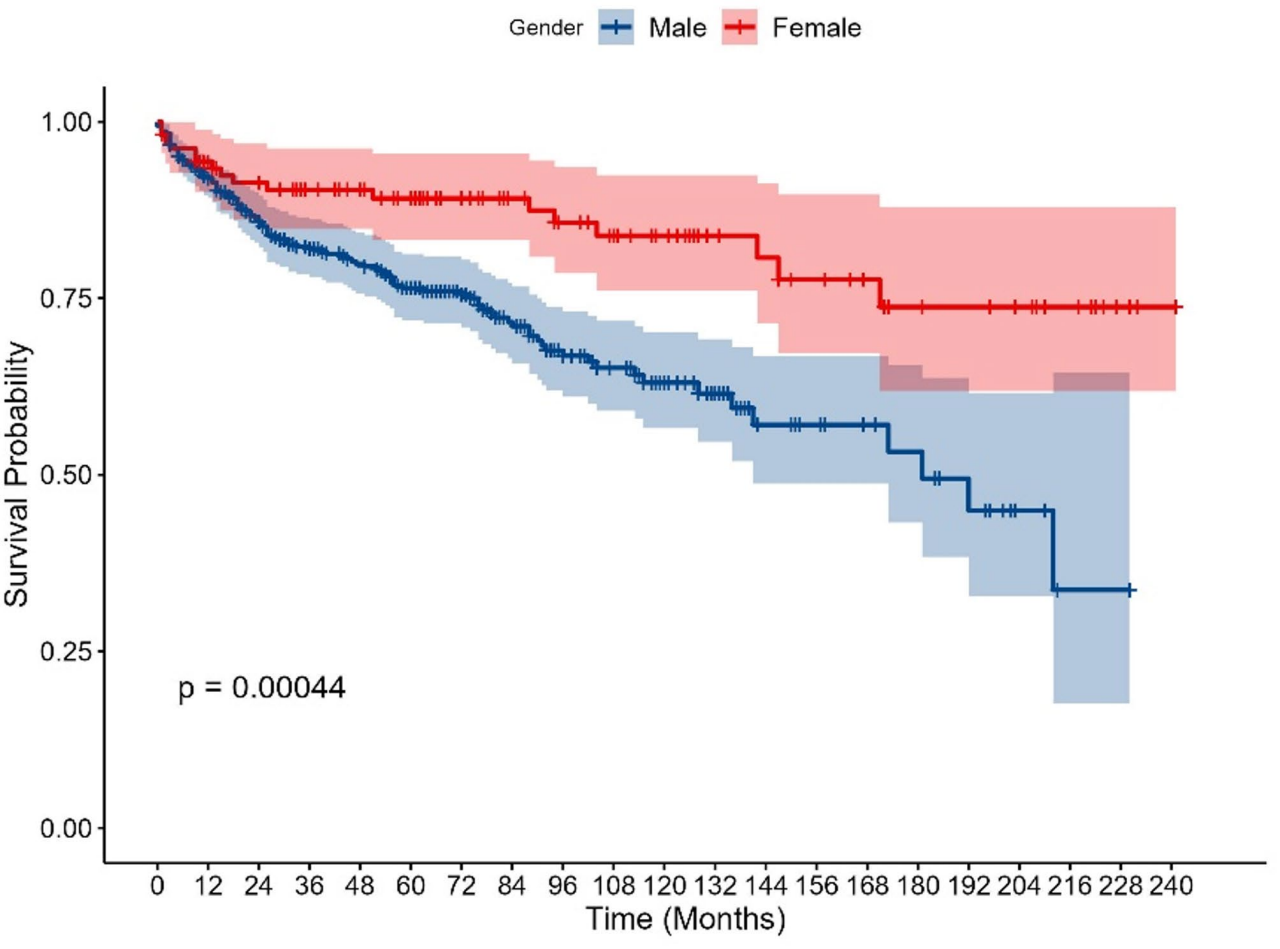
Kaplan–Meier survival curve by ‘gender’.

**Fig. 2:**
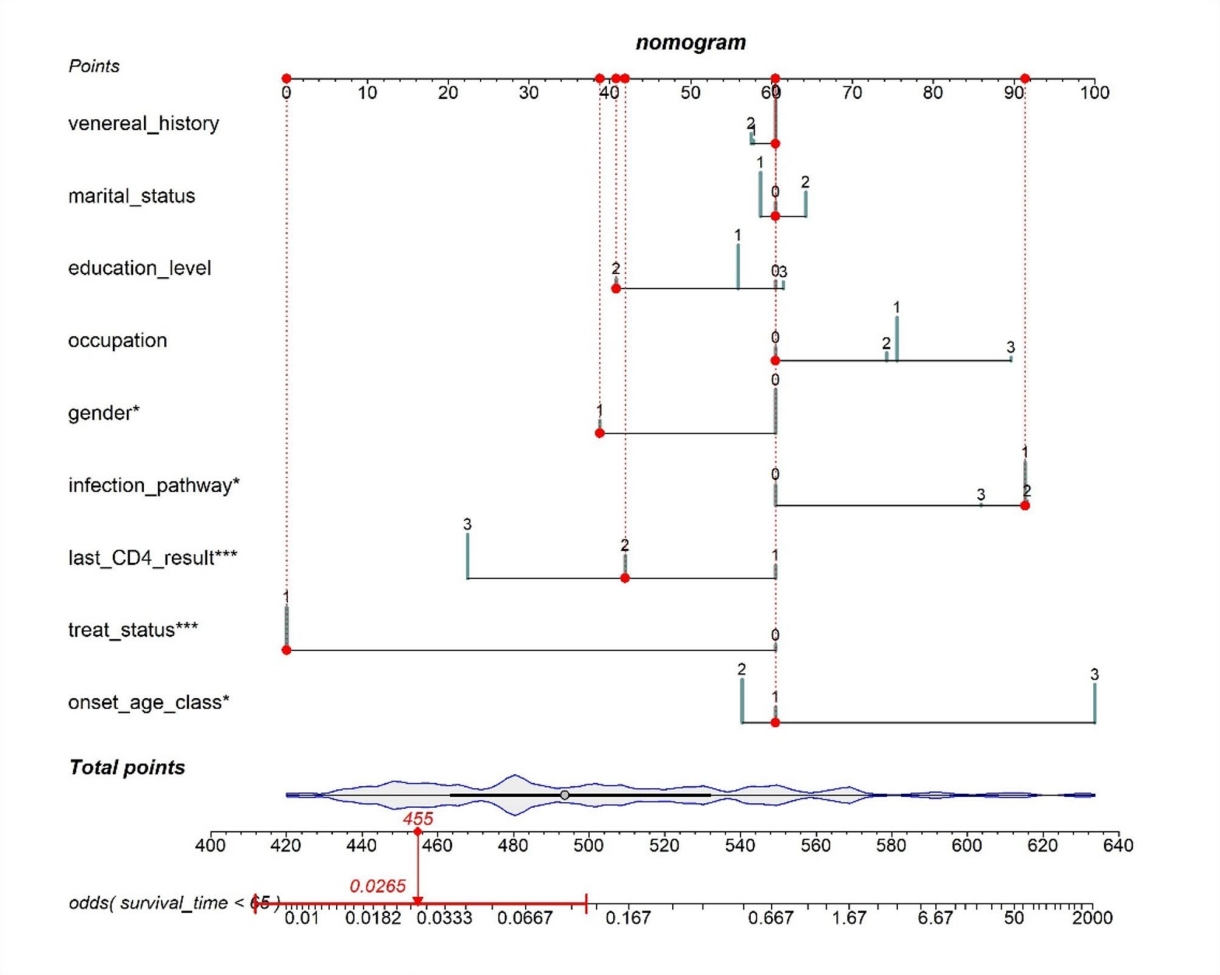
Nomogram of the multivariate cox proportional hazard model (the cumulative sum of the points scales corresponding to the different classifications of predictors yields total points, which maps the odds of the final predicted outcome).

**Fig. 3:**
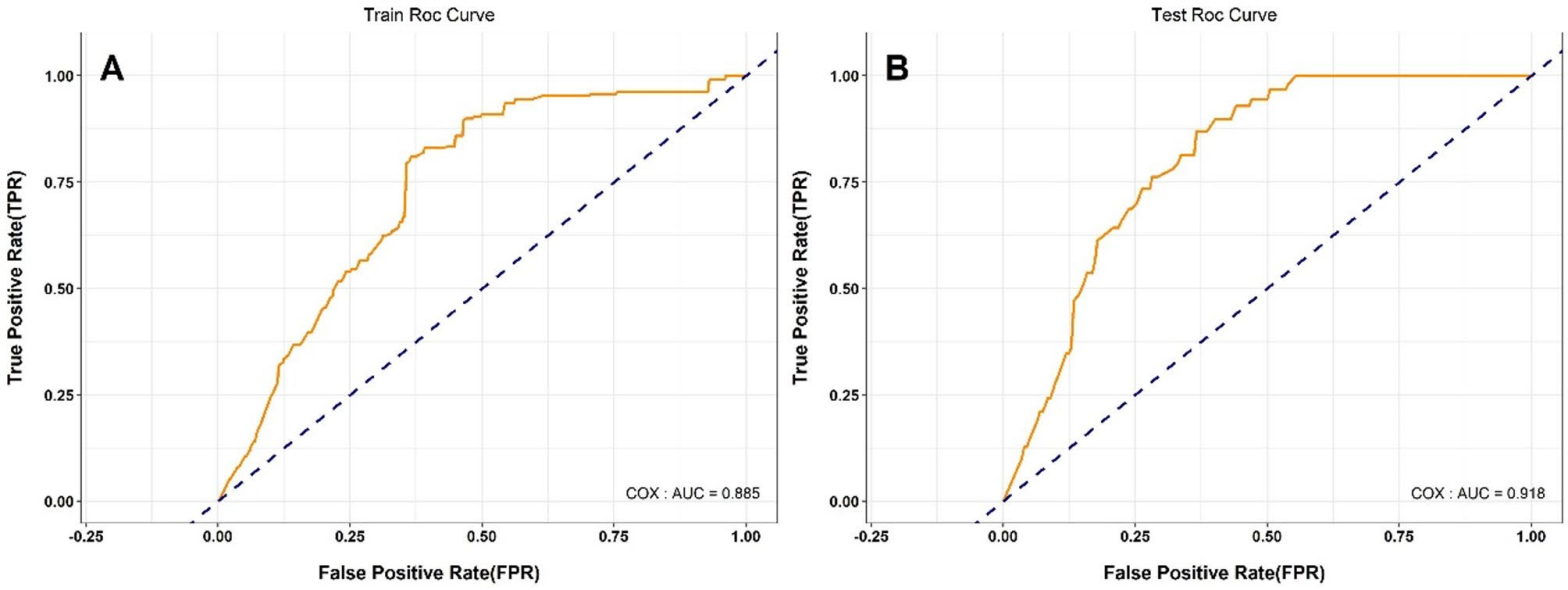
ROC curves for predicting survival rates in the training (**A**) and testing (**B**) cohorts using the multivariate cox proportional hazard model.

Table 2 presents the results of univariate and multivariate Cox regression analyses of risk factors for PLWH. The study conducted a Cox univariate analysis on nine variables, all of which had p-values less than 0.05, consistent with the assumptions of the proportional risk model. We plotted Kaplan-Meier survival curves for all variables to visualise the differences in survival between groups (Fig. 1 and Fig. S1-S8). In terms of gender, for example, there was a statistically significant difference in survival probability between males and females. Females consistently showed higher survival probability and slower decline in survival compared to males (Fig. 1). In addition, we performed correlation and multicollinearity analyses for the nine variables, which showed no high correlation or multicollinearity (Table S3 and Fig. S9). Further inclusion into the multivariate Cox proportional hazard model showed that gender, infection_pathway, last_CD4_result, treat_status, and onset_age_class were statistically significant. The Cox proportional hazard model was constructed based on the training cohort, resulting in a nomogram that can predict the survival rate of PLWH, as shown in Fig. 2. The nomogram integrates multiple independent predictors, and the total points scale can be obtained by calculating the points scale above the nomogram corresponding to different predictors to quantify the odds of predicting a specific clinical event below the nomogram. Receiver operating characteristic(ROC) analysis indicated that the model AUC value was 0.885 (Fig. 3A). Using the testing cohort to validate the Cox proportional hazard model, the model AUC value was 0.918 (Fig. 3B).

**Table 2:** Univariate and multivariate Cox regression analysis of risk factors in PLWH.

Variable	Univariate analysis OR (95% CI)	*P* Value	Multivariate analysis OR (95% CI)	*P* Value
Onset_age_class		<0.001		
< 40	Reference		Reference	
≥ 40, < 60	1.23 (0.46–3.28)	0.677	0.88 (0.31–2.46)	0.803
≥ 60	7.91 (3.22–19.47)	<0.001	3.51 (1.30–9.50)	0.013
Gender		<0.001		
Male	Reference		Reference	
Female	0.40 (0.24–0.68)	<0.001	0.50 (0.28–0.91)	0.024
Marital_Status		<0.001		
Unmarried	Reference			
Married with Spouse	0.62 (0.36–1.05)	0.074		
Discovered or Widowed	1.83 (1.10–3.04)	0.020		
Education_Level		<0.001		
Illiterate	Reference			
Junior High School and Below	0.40 (0.26–0.61)	<0.001		
High School/Technical Secondary School	0.12 (0.05–0.29)	<0.001		
College and above	0.14 (0.05–0.41)	<0.001		
Occupation		0.002		
Unemployed	Reference			
Farmer	2.15 (1.22–3.76)	0.008		
Laborer	0.69 (0.27–1.80)	0.447		
Business service and others	1.51 (0.58–3.92)	0.402		
Infection_pathway		<0.001		
Homosexual transmission	Reference		Reference	
Heterosexual transmission	5.57 (2.71–11.48)	<0.001	2.67 (1.16–6.14)	0.021
Bloodborne transmission	2.97 (1.15–7.64)	0.024	2.69 (0.93–7.81)	0.068
Other routes	4.65 (1.22–17.72)	0.024	2.25 (0.54–9.36)	0.267
Venereal_history		0.013		
None	Reference			
Yes	0.97 (0.45–2.10)	0.948		
Unknown	0.40 (0.22–0.75)	0.004		
Last_CD4_result		<0.001		
< 200	Reference		Reference	
≥ 200, < 400	0.41 (0.27–0.63)	<0.001	0.55 (0.35–0.87)	0.010
≥ 400	0.14 (0.09–0.23)	<0.001	0.30 (0.18–0.49)	<0.001
ART_treatment_status		<0.001		
No	Reference		Reference	
Yes	0.12 (0.08–0.18)	<0.001	0.15 (0.09–0.23)	<0.001

### Machine learning model prediction results

**Fig. 4:**
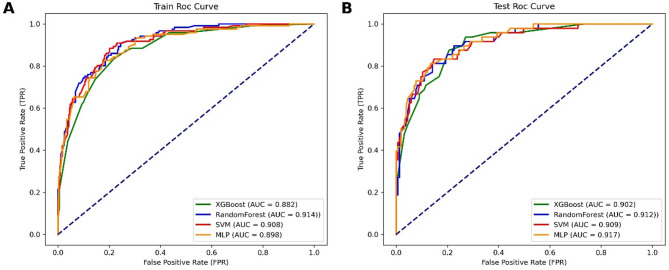
ROC curves of four machine learning models for predicting survival rates in the training (**A**) and testing (**B**) cohorts.

**Fig. 5:**
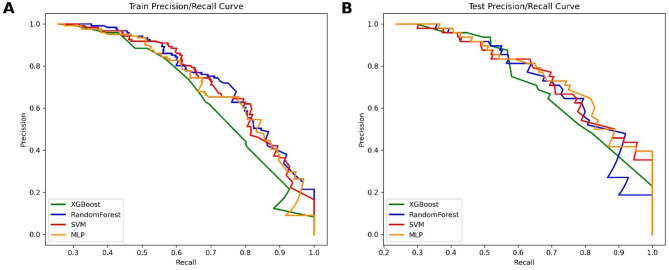
Precision-recall curves of four machine learning models for predicting survival rates in the training (**A**) and testing (**B**) cohorts.

**Fig. 6:**
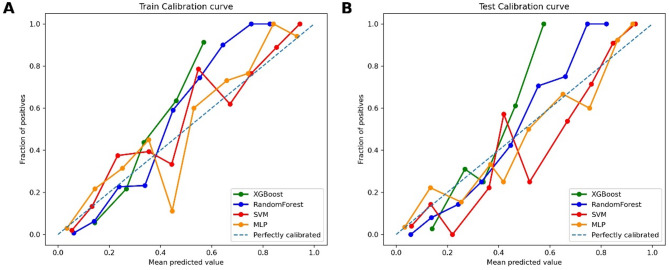
Calibration curves of four machine learning models for predicting survival rates in the training (**A**) and testing (**B**) cohorts.

Using nine feature variables obtained from univariate Cox regression analysis, four machine learning models were constructed (Table 3). Based on the training cohort, the model prediction results were as follows: the XGBoost model had an AUC value of 0.882, the RF model had an AUC value of 0.914, the SVM model had an AUC value of 0.908, and the MLP model had an AUC value of 0.898 (Fig. 4A). Internal validation was performed on the constructed models using the testing cohort, with the following results: the XGBoost model had an AUC value of 0.902, the RF model had an AUC value of 0.912, the SVM model had an AUC value of 0.909, and the MLP model had an AUC value of 0.917 (Fig. 4B). The RF model stood out among all models, followed by the SVM model. Given that the survival and death results in the cohort did not meet a 1:1 ratio, Precision-Recall curves were needed to compensate for the limitations of the AUC value, providing a fuller explanation of model performance. A comprehensive assessment showed that the RF model had higher precision than the other models, indicating stronger predictive capabilities (Fig. 5). Calibration curves demonstrated the relationship between the model predictions and actual outcomes. In the training cohort, all models’ calibration curves were relatively ideal. In the testing cohort, the RF model’s curve was closer to the perfectly calibrated line in most areas, indicating better calibration (Fig. 6). Thus, the performance of the RF model was more outstanding.

**Table 3:** Performance metrics of four machine learning models in the training and testing cohort.

Model	Training					Test				
	AUC	Accuracy	Precision	Recall	F1	AUC	Accuracy	Precision	Recall	F1
XGboost	0.882	0.784	0.913	0.174	0.292	0.902	0.813	1.000	0.208	0.345
RF	0.914	0.848	0.836	0.504	0.629	0.912	0.862	0.794	0.562	0.659
SVM	0.908	0.863	0.798	0.620	0.698	0.909	0.862	0.727	0.667	0.696
MLP	0.898	0.865	0.782	0.653	0.712	0.917	0.877	0.756	0.708	0.731

*XGboost* eXtreme gradient boosting, *RF* random forest, *SVM* support vector machine, *MLP* multilayer perceptron.

### Macro analysis of SHAP values

**Fig. 7:**
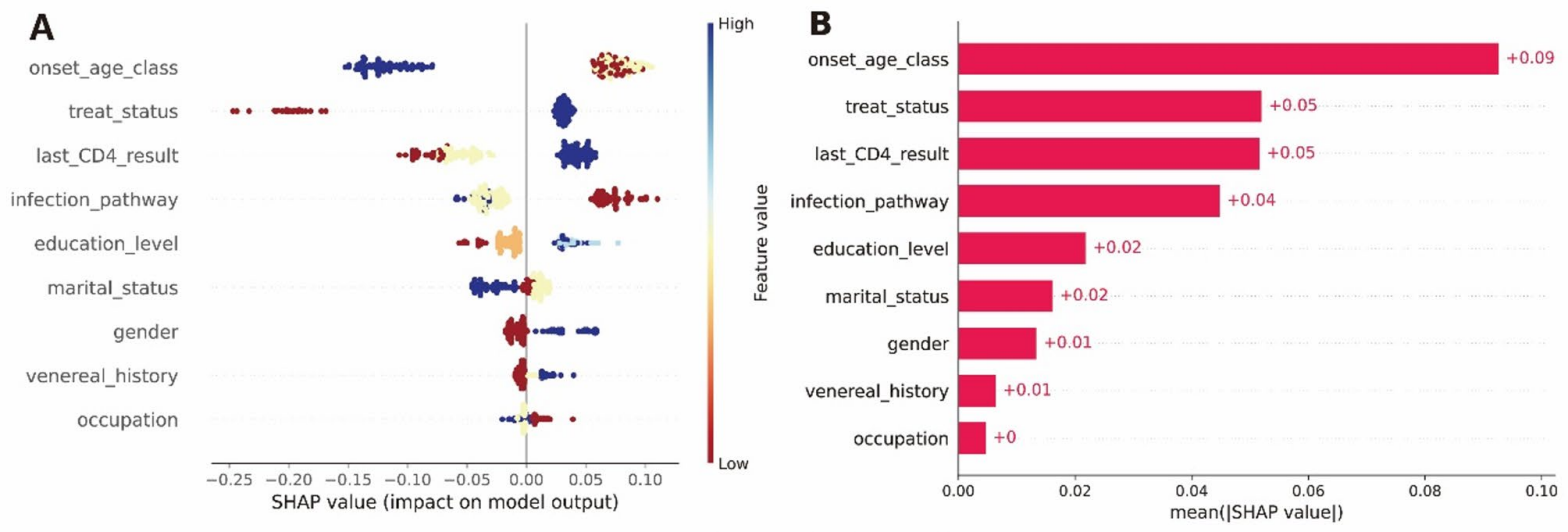
Importance ranking of feature variables by SHAP values in the RF model (**A** each dot represents a sample. The horizontal axis shows the SHAP value of the feature. A positive SHAP value indicates a positive impact, while a negative SHAP value indicates a negative impact. **B** Obtained by ranking based on the average absolute value of feature importance for each variable).

Due to the black-box nature of machine learning models, it is challenging to interpret the relationships between variables. SHAP values help explain model outputs by assigning importance values to features. Estimating SHAP values can facilitate a better understanding of the workings of machine learning models. Based on the RF algorithm model, SHAP values are calculated and used to rank all variables, indicating the extent of different variable features’ impact on the survival status of PLWH (Fig. 7). Blue indicates higher feature values, red represents lower feature values, and yellow indicates feature values close to the mean. Estimating SHAP values can facilitate a better understanding of the workings of machine learning models. It can be observed that the larger the SHAP value of a variable, the better the survival outcome and the lower the risk of death for PLWH (Fig. 7A). Using the ‘onset_age_class’ variable as an example, the blue color indicates higher feature values, representing an older age at diagnosis. A negative SHAP value suggests a negative impact on the survival rate of PLWH. Figure 7B further displays the ranking of feature variable importance.

### Microanalysis of SHAP values

**Fig. 8:**
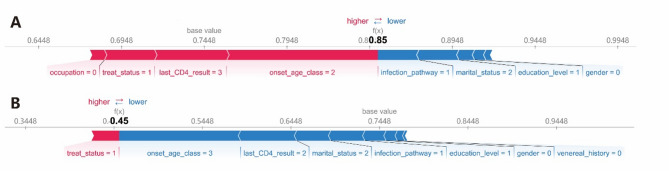
Feature impact diagrams for two outcomes in the RF model (**A** force plot for PLWH who were alive at the time of study enrollment. **B** force plot for PLWH who were deceased at the time of study enrollment).

A force plot is a method used in SHAP value analysis to analyze influencing factors on an individual basis, providing explanations for predictions on a single sample. Red indicates a positive contribution, while blue indicates a negative contribution. The base value represents the constant in the explanatory model. The study created force plots for one surviving PLWH (Fig. 8A) and one deceased PLWH (Fig. 8B). In the force plot for the surviving PLWH, the ‘onset_age_class’ between 40 and 60 had the greatest positive impact on survival, followed by the last CD4 count being greater than or equal to 400, currently receiving ART, and unemployed status. Heterosexual transmission had the most significant negative impact on survival, followed by divorced or widowed marital status. In the force plot for the deceased PLWH, ‘onset_age_class’ greater than or equal to 60 had the largest negative impact on survival, followed by the last CD4 count between 200 and 400, divorced or widowed, heterosexual transmission, and education level of junior high school or below. Receiving ART treatment had the most significant positive impact on survival.

## Discussion

According to the World Health Organization, HIV remains a major global public health issue to date. ART schemes for treating human HIV infections have reduced the HIV viral load in PLWH to undetectable levels and restored CD4^+^T cell counts to normal levels, significantly reducing AIDS mortality^24^. Over the past years, the survival rate of the PLWH population has significantly improved, and there has been a shift in the causes of death from AIDS-related to non-AIDS-related^25^. Although current treatments have significantly extended the lifespan of PLWH, early prediction of disease progression, identification of high-risk factors, and early intervention measures to mitigate risks can further improve the quality of life and survival time of PLWH. Therefore, survival analysis is particularly important for accurately identifying risk factors affecting survival rates.

Machine learning models possess strong feature extraction capabilities and excel in personalized prediction, finding broad applications in survival analysis across various diseases. Unlike traditional survival analysis methods, survival prediction models developed using machine learning techniques achieve higher accuracy. Most traditional statistical methods are limited in efficiency and struggle to capture complex nonlinear relationships. For example, the Cox proportional hazard model, which is typically suited for low-dimensional data, assumes linear relationships among variables. In the medical field, data is often voluminous, high-dimensional, and complex, frequently requiring high-performance computing to handle large-scale data. Machine learning algorithms are capable of processing high-dimensional data and excel at capturing complex nonlinear relationships and interactions between variables. A meta-analysis has shown that machine learning models perform better in predicting the survival of PLWH^4^.

We successfully developed a high-precision machine learning-based model for predicting the long-term survival risk of PLWH. In this study, we quantitatively compared traditional statistical analysis and machine learning methods for their prognostic ability and accuracy in predicting the survival of PLWH, based on a cohort with approximately 20 years of monitoring. We conducted multivariate Cox proportional hazard analysis and created nomograms to better understand the model, although nomograms focus more on the overall model interpretation. By running machine learning code, we tested four machine learning models: XGBoost, RF, SVM, and MLP. After parameter tuning and five-fold cross-validation, we found that the RF model was the most successful, followed by the SVM model. The XGBoost model and MLP model performed well in the testing cohort but were less ideal in the training cohort, with both showing substantial risk of model overfitting. In addition, we also trained DeepSurv model, DeepHit model, and Random survival forest model(Table S4). However, similar to the deep learning based MLP model, DeepSurv model may have the risk of overfitting due to the more complex model relative to the data(Fig S10). The DeepHit model, and Random survival forest model have average training results(Fig S11 and Fig S12). The performance of the multivariate Cox proportional hazard model was between that of the XGBoost and MLP models.

Based on the results of model comparisons, we successfully developed an RF machine learning model for predicting the survival rate of PLWH after diagnosis. Unlike traditional approaches reliant on single indicators or empirical judgments, this method has the potential to provide healthcare professionals with a dynamic and individualized risk assessment tool. Our model integrates multiple prognostic factors to generate continuous survival risk scores for PLWH, enabling direct application in clinical settings. The model also demonstrates the capability to identify high-risk individuals frequently overlooked by traditional methods, facilitating early and aggressive intervention to prevent the risk of deterioration. In practical clinical implementation, we believe that data-driven decision support systems will facilitate more precise allocation of medical resources, thus enhancing the management efficiency and treatment outcomes of PLWH.

In choosing machine learning models, one often needs to consider both the model’s interpretability and prediction accuracy^26^. The RF model, while having lower interpretability, offers high prediction accuracy^27^. This study utilized SHAP values to enhance the interpretability of the RF model^26^. Compared to nomograms, SHAP value analysis can also focus on explaining outcomes for PLWH with different prognoses. We found that the age at diagnosis, receiving ART or not and recent CD4^+^T cell count significantly impact PLWH for both survival and death outcomes. Therefore, we can identify age at diagnosis, receiving ART or not and recent CD4^+^ counts as core variables affecting long-term survival. These results could prompt clinicians to routinely and qualitatively monitor high-weighted prognostic factors after a patient is diagnosed with the virus. Because Force plots can be used to enable dynamic risk stratification and are easy to understand and use effectively, they can facilitate more effective tracking, follow-up, and management of PLWH by clinicians. In addition, PLWH survival prediction models developed based on machine learning models can help drive access to care. In areas where expert experience is lacking, the models can help primary care providers make better decisions.

There are still some limitations in our study. First, we only selected the PLWH population from Hebi, Henan Province, China, for our research. The survival prediction model may show some deviation in prediction results in other regions. Although the distribution of the characteristics of PLWH in Hebi is similar to the overall situation in Henan Province, and the survival of PLWH in Henan Province can represent the overall situation in China to a certain extent, further studies need to carry out a national multicentre epidemiological survey of PLWH to improve the generalizability of the model. Secondly, our study is a retrospective cohort study, and further prospective cohort studies are needed to validate the model.

## Conclusion

This study is the first to compare the performance and effectiveness of the Cox proportional hazard model and four machine learning models in predicting the survival of PLWH. By using baseline information and clinical factors of PLWH for comprehensive analysis, it was found that the predictive performance of machine learning models surpasses that of traditional Cox survival prediction models. The survival prediction model based on the RF model is considered to have the best predictive effectiveness. Then, this paper constructs and establishes an internal validation cohort to validate the RF-based survival prediction model for PLWH and evaluates model performance using AUC values, Precision-Recall curves, and calibration curves. Additionally, nomograms and SHAP values were used to further interpret the model, facilitating an understanding of the impact mechanisms of feature variables.

## Supplementary Information

Below is the link to the electronic supplementary material.

Supplementary Material 1

## Data Availability

All data generated or analyzed during this study are included in this article. Further inquiries can be directed to the corresponding author.
