# Supplementary material for: Survival prediction models for people living with HIV based on four machine learning models
Source: Sci Rep. 2025 Aug 25;15:31256. doi: 10.1038/s41598-025-16479-3 (PMC12378378; doi:10.1038/s41598-025-16479-3)
Supplement: Supplementary file 1 — Supplementary Material 1 [file 41598_2025_16479_MOESM1_ESM.docx]

***Supplementary Materials***


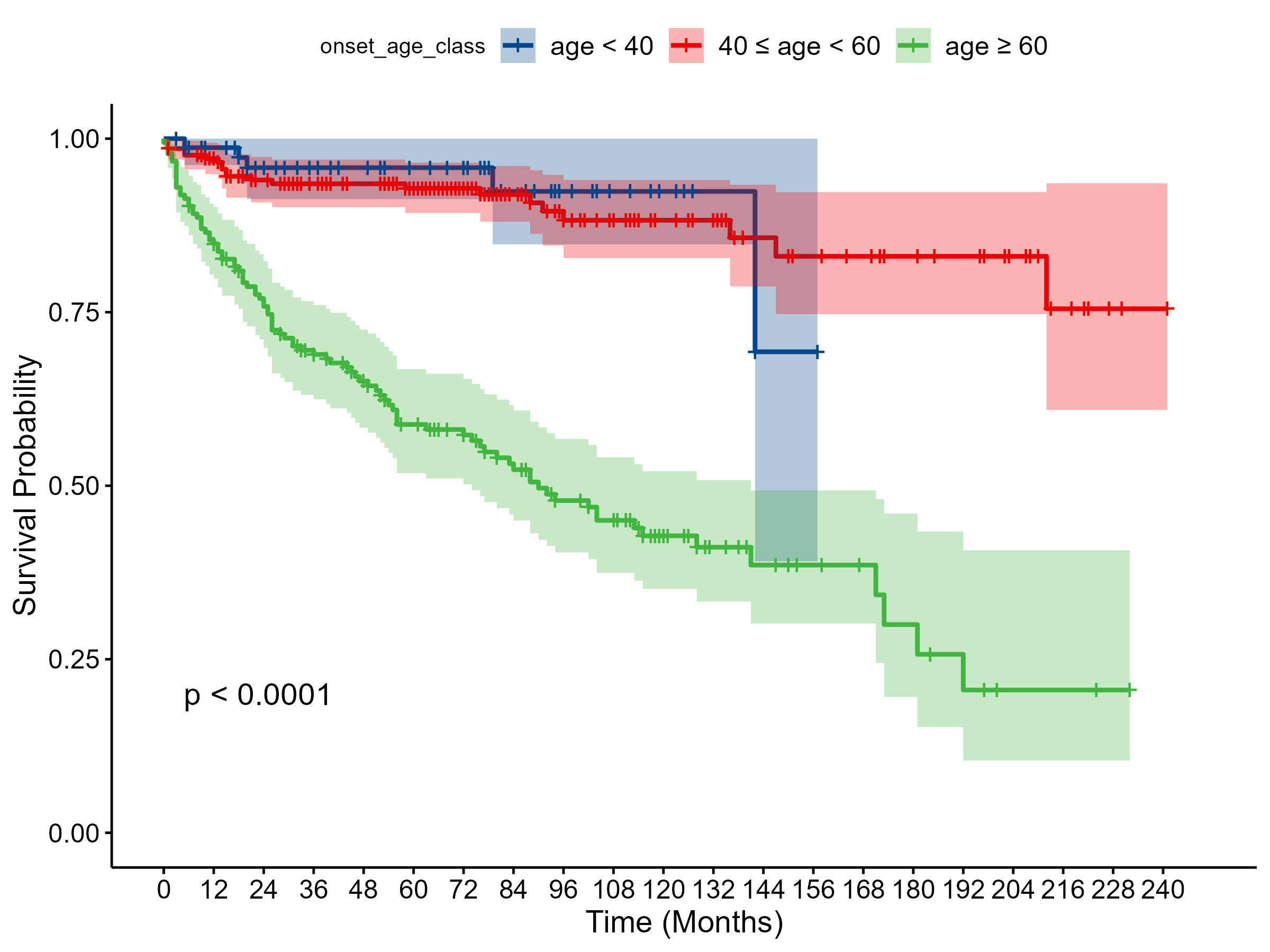


Figure S1 Kaplan-Meier Survival Curve by ‘onset_age_class’


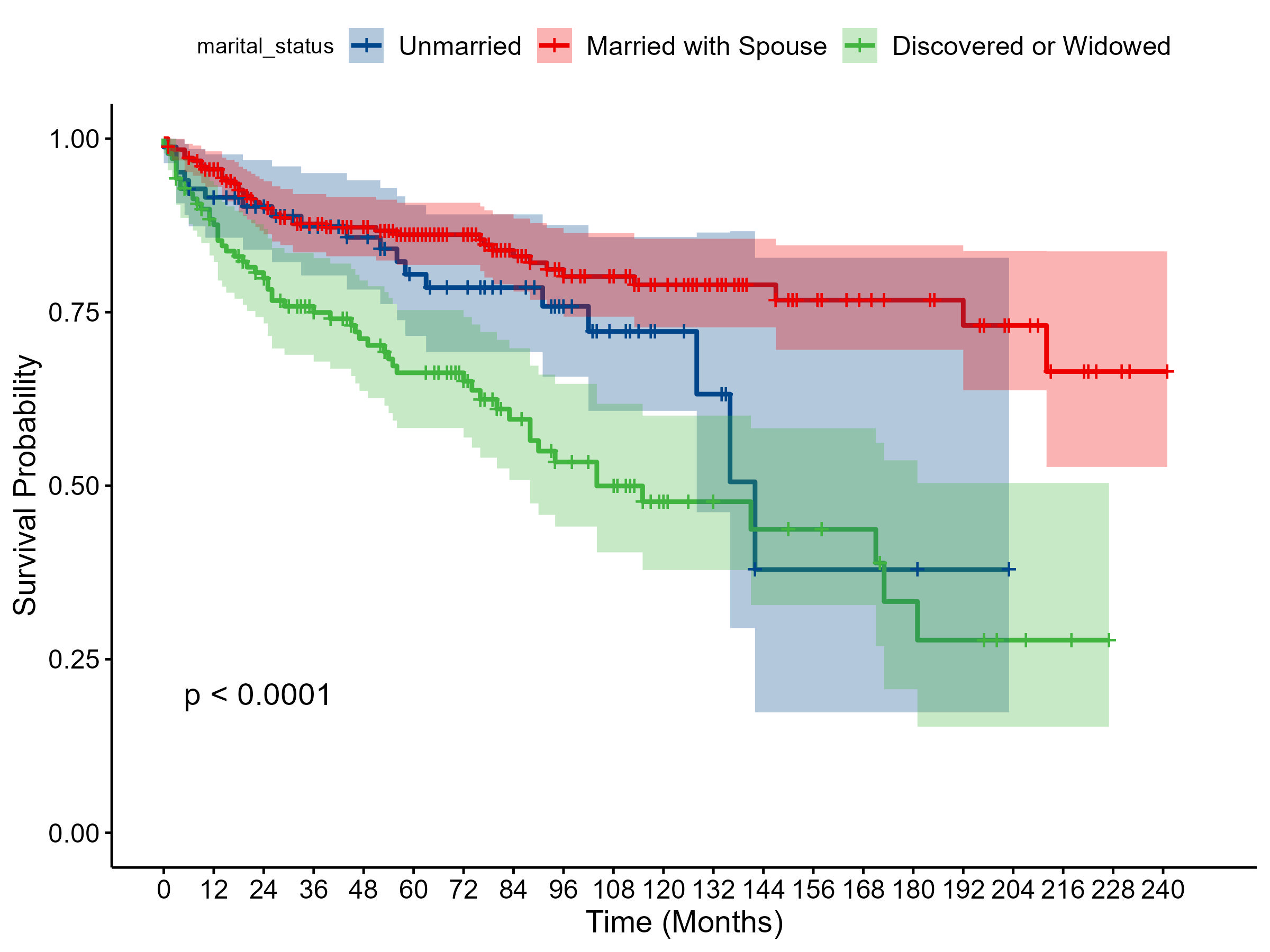


Figure S2 Kaplan-Meier Survival Curve by 'marital_status'


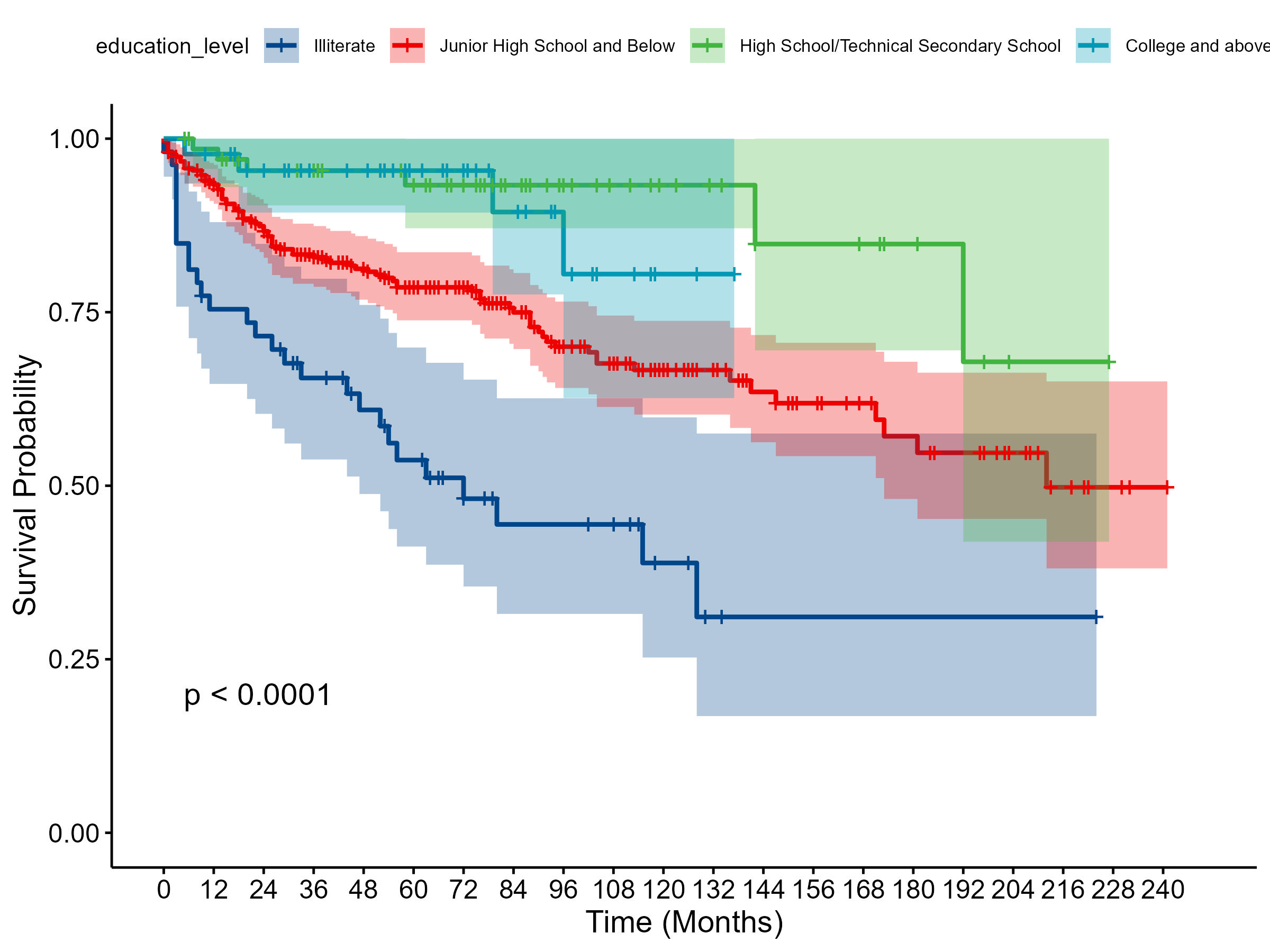


Figure S3 Kaplan-Meier Survival Curve by 'education_level'


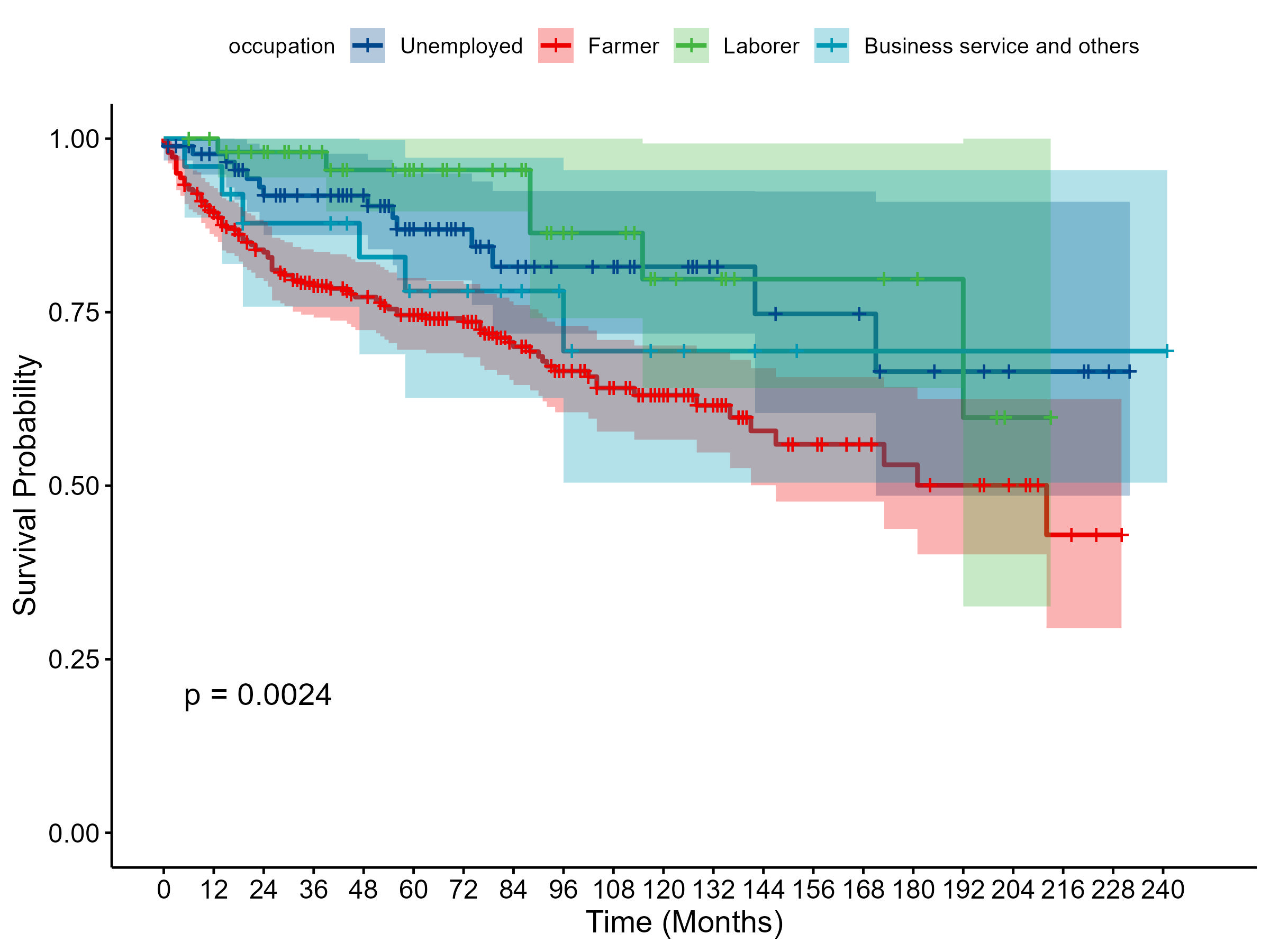


Figure S4 Kaplan-Meier Survival Curve by 'occupation'


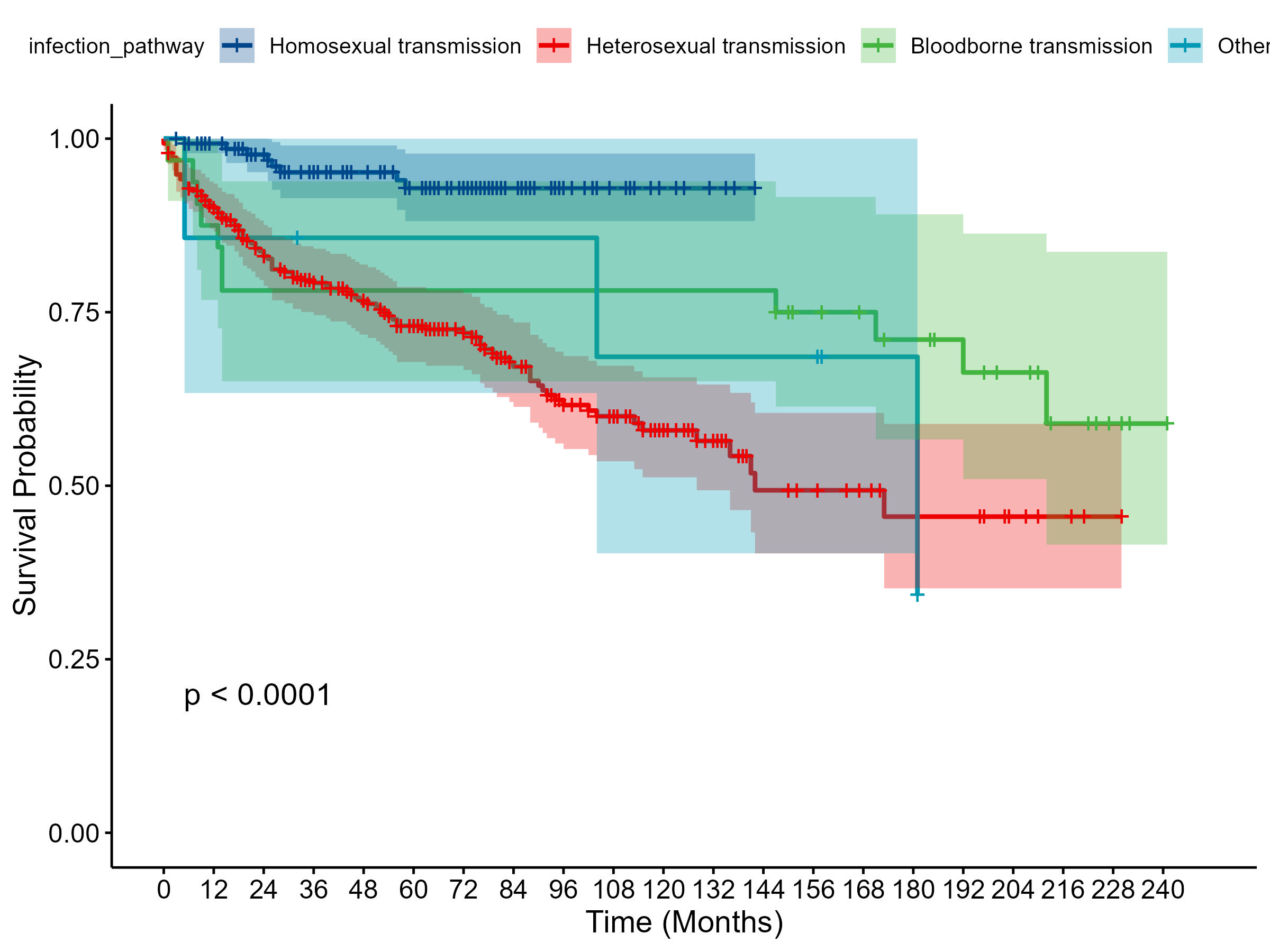


Figure S5 Kaplan-Meier Survival Curve by 'infection_pathway'


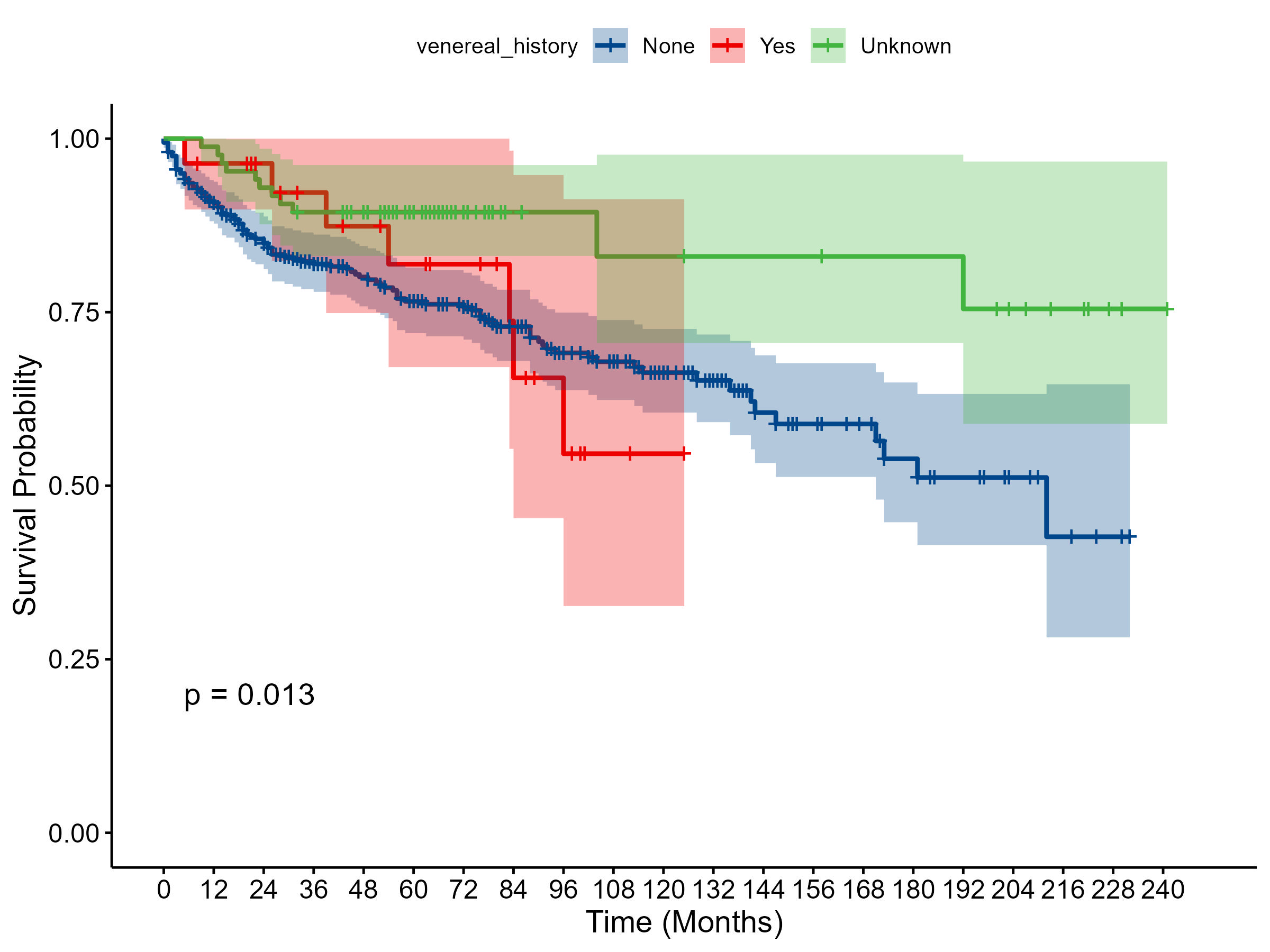


Figure S6 Kaplan-Meier Survival Curve by 'venereal_history'


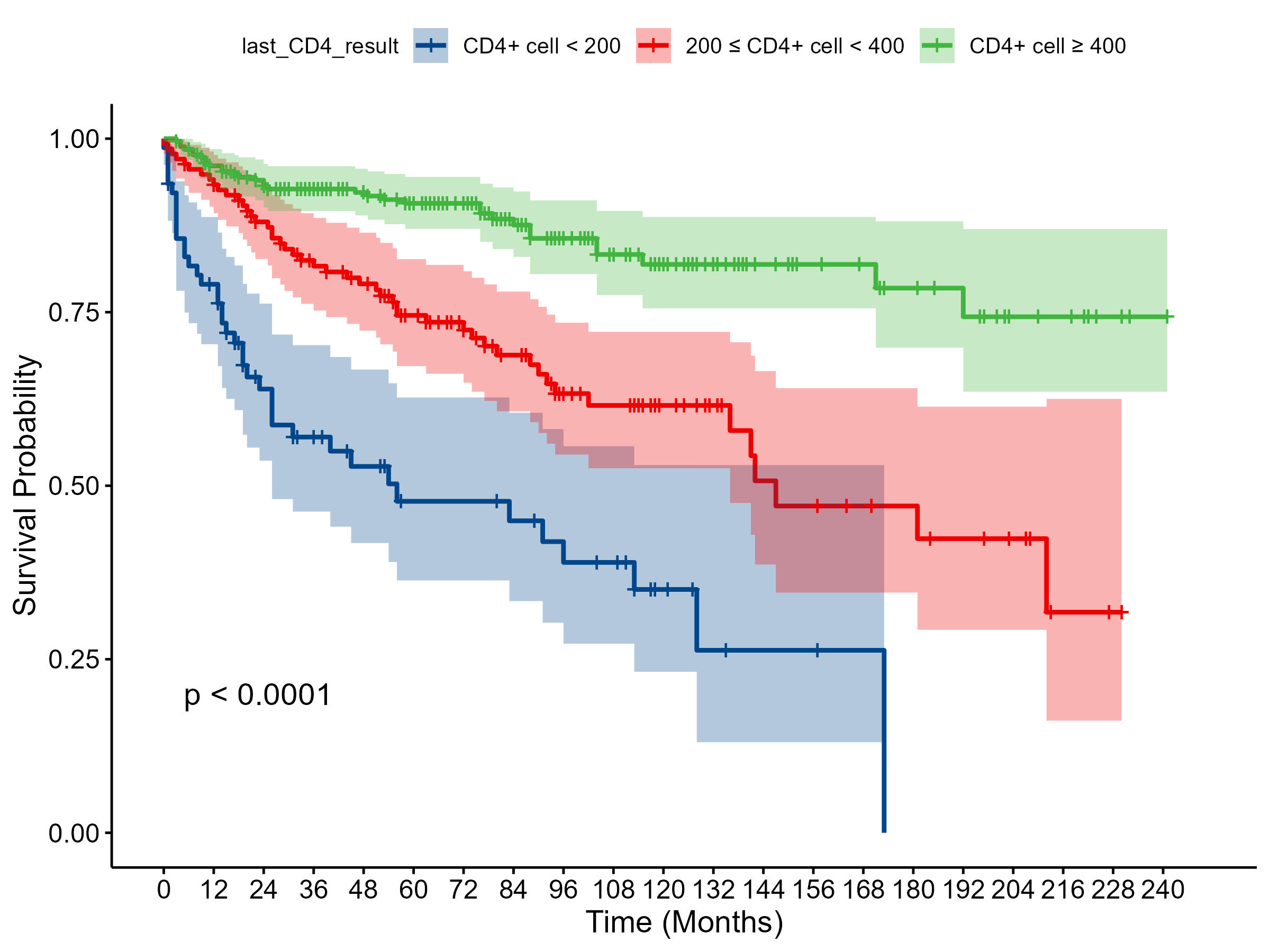


Figure S7 Kaplan-Meier Survival Curve by 'last_CD4_result'


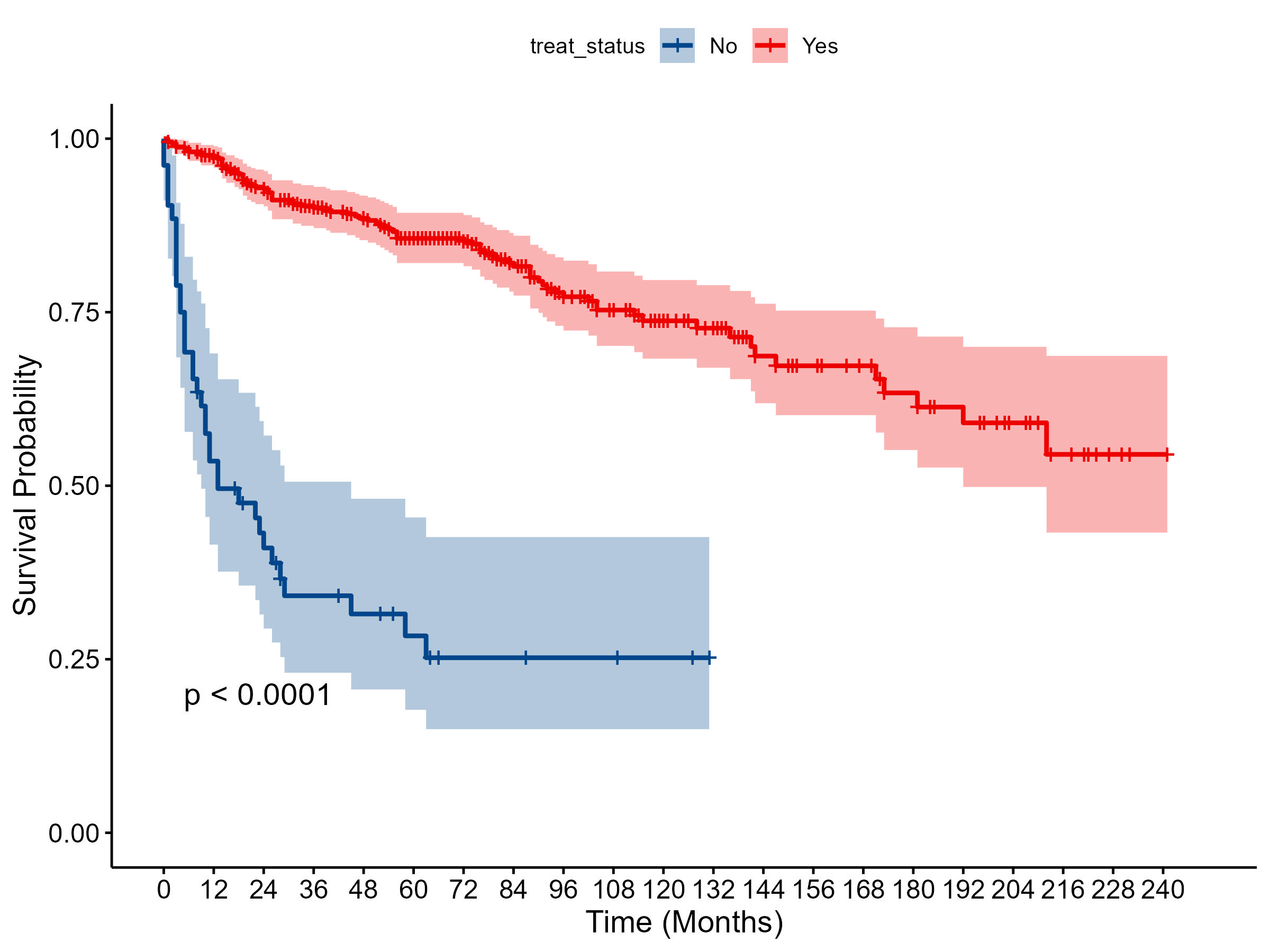


Figure S8 Kaplan-Meier Survival Curve by 'treat_status'


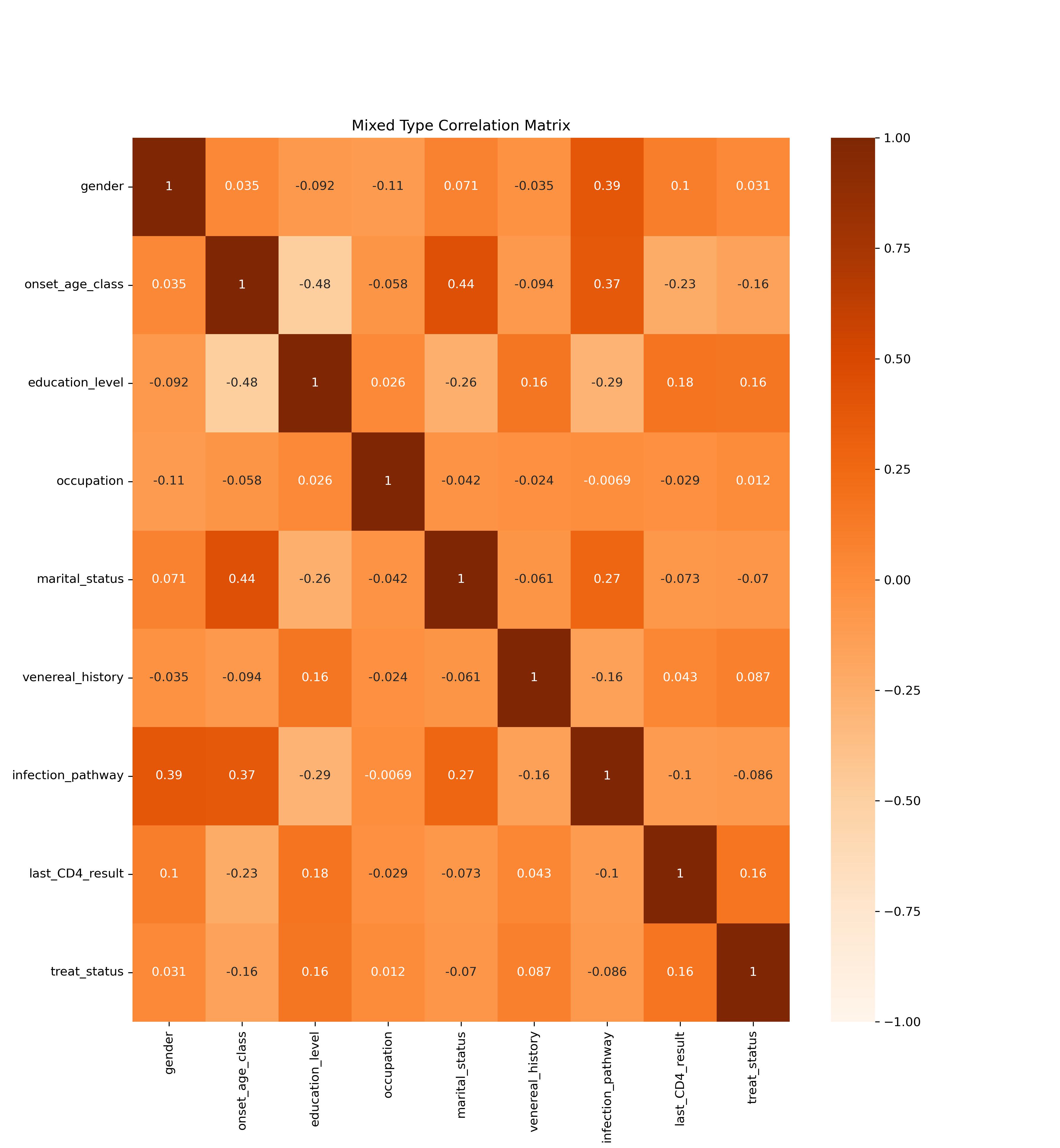


Figure S9 Correlation Matrix


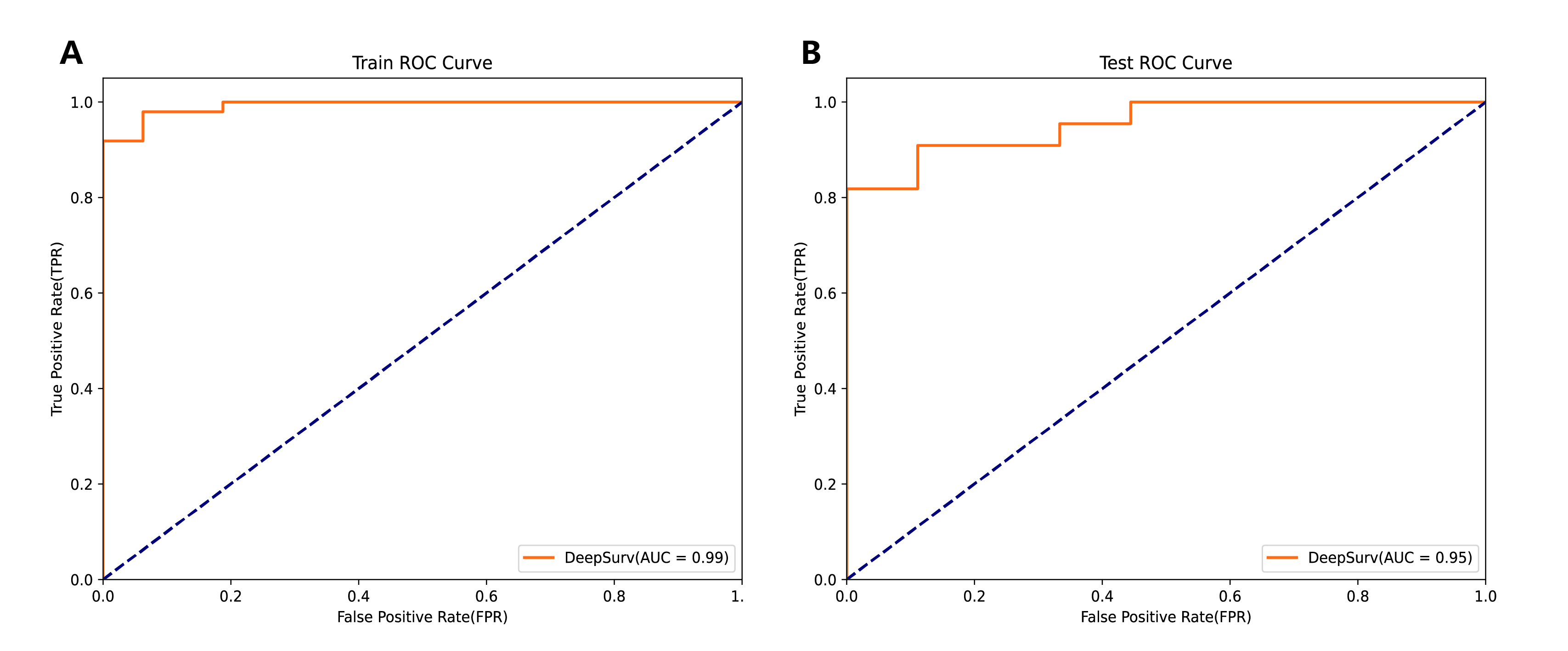


Figure S10 ROC Curves of DeepSurv model for Predicting Survival Rates in the Training(A) and Testing(B) Cohort


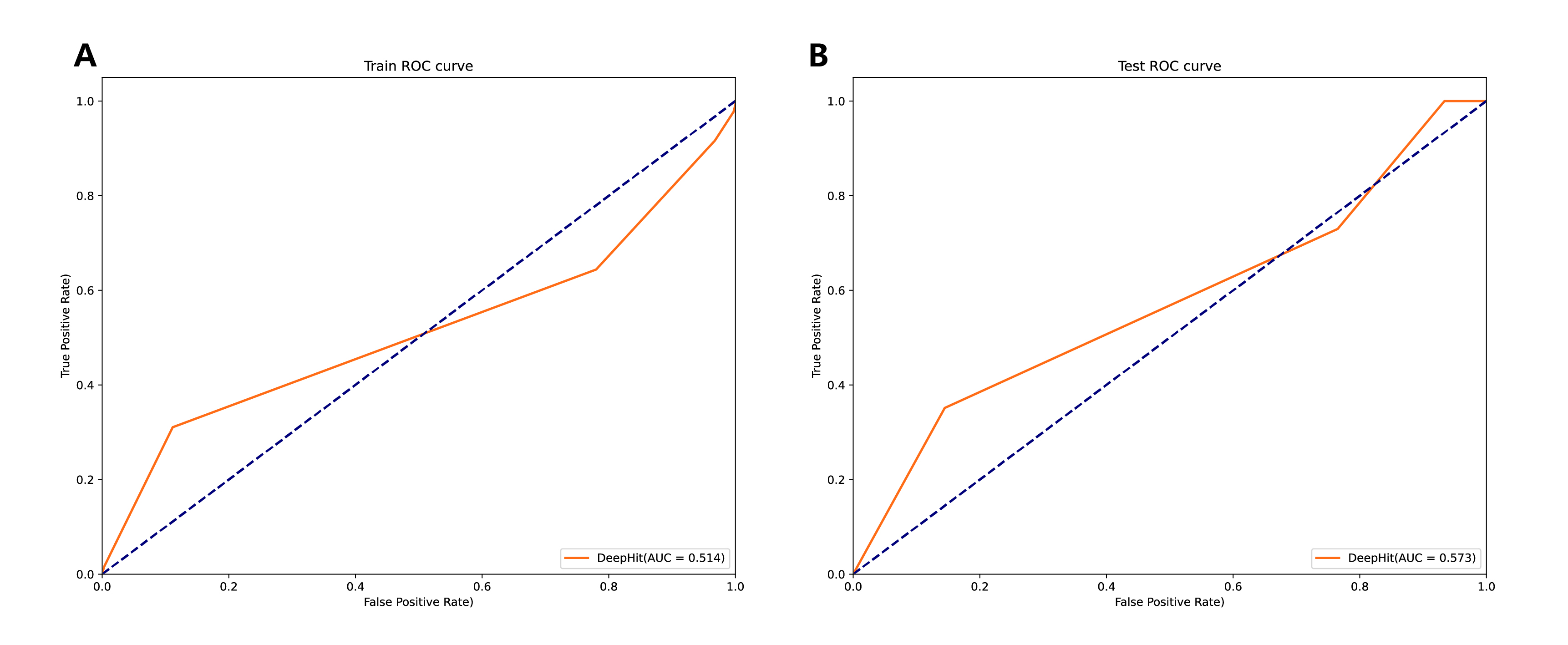


Figure S11 ROC Curves of DeepHit model for Predicting Survival Rates in the Training(A) and Testing(B) Cohort


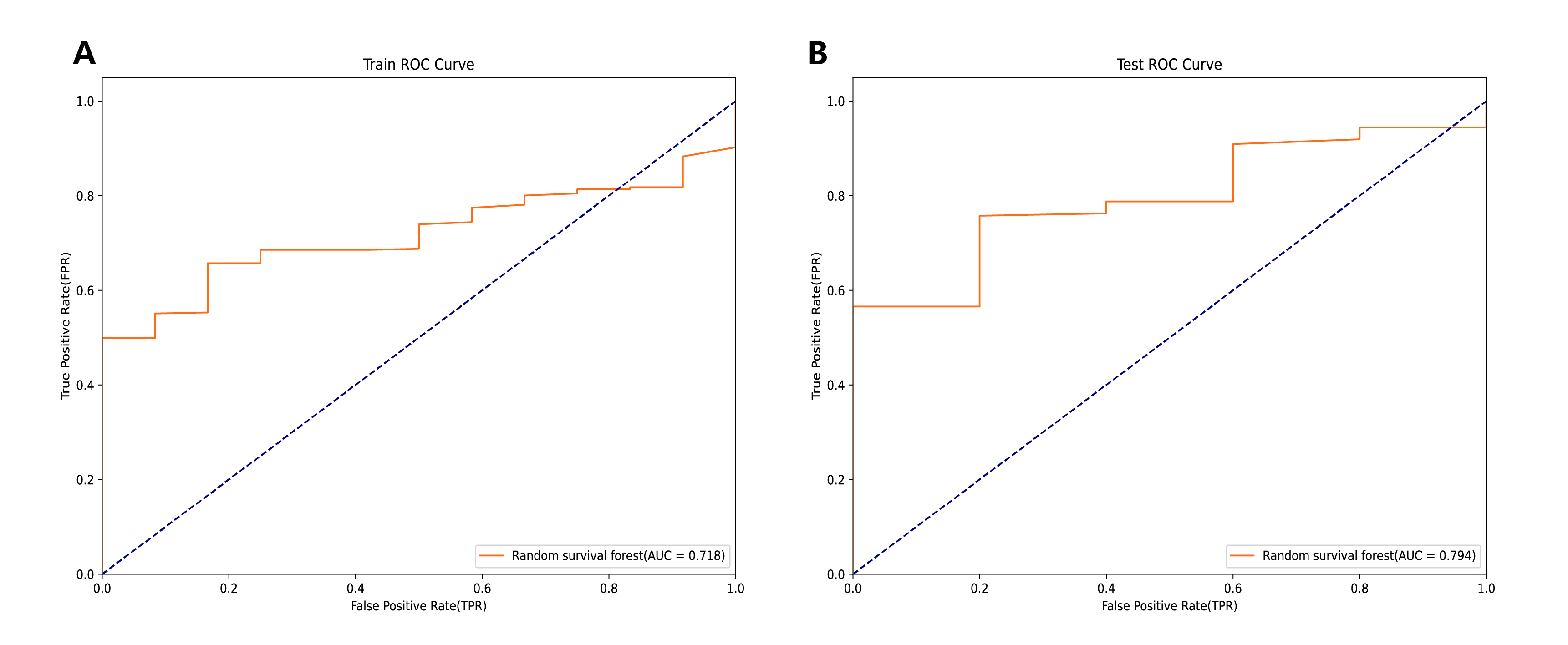


Figure S12 ROC Curves of Randomsurvival forest model for Predicting Survival Rates in the Training(A) and Testing(B) Cohort

Table S1 Variable Assignment

| Primary variables | Secondary variables | Assignment |
| --- | --- | --- |
| onset_age_class | age < 40 | 1 |
|  | 40 ≤ age < 60 | 2 |
|  | age ≥ 60 | 3 |
| gender | Male | 0 |
|  | Female | 1 |
| marital_status | Unmarried | 0 |
|  | Married with Spouse | 1 |
|  | Discovered or Widowed | 2 |
| education_level | Illiterate | 0 |
|  | Junior High School and Below | 1 |
|  | High School/Technical Secondary School | 2 |
|  | College and above | 3 |
| occupation | Unemployed | 0 |
|  | Farmer | 1 |
|  | Laborer | 2 |
|  | Business service and others | 3 |
| infection_pathway | Homosexual transmission | 0 |
|  | Heterosexual transmission | 1 |
|  | Bloodborne transmission | 2 |
|  | Other routes | 3 |
| venereal_history | None | 0 |
|  | Yes | 1 |
|  | Unknown | 2 |
| last_CD4_result | CD4+ cell < 200 | 1 |
|  | 200 ≤ CD4+ cell < 400 | 2 |
|  | CD4+ cell ≥ 400 | 3 |

Table S2 Hyper-parameter tuning

| Model | Hyper-parameter tuning |
| --- | --- |
| Xgboost | objective='binary:logistic',  booster='gbtree',  nthread=4,  seed=520,  n_estimators=9,  max_depth=4,  min_child_weight=7,  colsample_bytree=1.0,  subsample=1.0,  reg_alpha=0.5,  reg_lambda=1.5,  learning_rate=0.1 |
| Random Forest | criterion='entropy',  n_estimators=200,  max_features=2,  max_depth=4,  min_samples_split=8,  min_samples_leaf=1,  min_impurity_decrease=0.001,  random_state=520,  oob_score=True |
| SVM | kernel='rbf',  random_state=520,  C=1,  gamma=0.1,  probability=True |
| MLP | random_state=520,  max_iter=700,  solver='sgd',  learning_rate_init=0.1,  hidden_layer_sizes=(13,2,4),  activation='relu',  alpha=0.001,  early_stopping=True,  learning_rate='adaptive',  tol=1e-4,  batch_size='auto',  momentum=0.9,  nesterovs_momentum=True |
| DeepSurv | hidden_layers=[64,32],  random_state=520,  dropout_rate=0.5,  activation='relu',  batch_size=32,  num_epochs=300,  criterion=CoxPHLoss(),  optimizer=optim.Adam(model.parameters(),lr=0.001) |
| DeepHit | shared_layers=[128,64],  cause_specific_layers=[32],  dropout_rate=0.2  criterion=DeepHitLoss(alpha=0.5)  optimizer=optim.Adam(model.parameters(),lr=0.001),  num_epochs=300 |
| Random Survival Forest | n_estimators=100,  min_samples_leaf=15,  max_features="sqrt",  random_state=520,  n_jobs=-1 |

*Model defaults are used for parameters not listed

Table S3 Variance inflation factors(VIF)

| Variable | VIF |
| --- | --- |
| onset_age_class | 1.661 |
| infection_pathway | 1.375 |
| education_level | 1.361 |
| marital_status | 1.291 |
| gender | 1.227 |
| last_CD4_result | 1.099 |
| treat_status | 1.058 |
| venereal_history | 1.040 |
| Occupation | 1.030 |

Table S4 C-index of Deepsurv, DeepHit, and Random survival forest models

| Metrics | Deepsurv | DeepHit | Randomsurvivalforest |
| --- | --- | --- | --- |
| C-index in trainset | 0.912 | 0.441 | 0.863 |
| C-index in testset | 0.859 | 0.389 | 0.879 |
| AUC in trainset | 0.992 | 0.514 | 0.718 |
| AUC in testset | 0.956 | 0.573 | 0.794 |
